# Evaluation of the Personality Disorder Positive Outcomes Programme (PDPOP) in general practice: an evaluation

**DOI:** 10.3399/BJGPO.2024.0196

**Published:** 2025-07-02

**Authors:** Hayley Trueman, Matt Williams, Robin Schafer, Fiona Blyth

**Affiliations:** 1 Patient Safety Team, Oxford Academic Health Science Network, Oxford, UK; 2 Oxford Health NHS Foundation Trust, Oxford, UK

**Keywords:** general practice, personality disorders, education and standards, primary health care

## Abstract

**Background:**

GPs and primary care services have been identified as crucial to the care of people with personality disorder. Individuals living with personality disorder frequently face stigma and difficulties when accessing health care. Primary care staff often describe feeling demoralised, incompetent, hurt, or angry after difficult interactions with patients.

**Aim:**

To evaluate the effect of Personality Disorder Positive Outcomes Programme (PDPOP) training delivered to 10 GP practices in 2022–2023.

**Design & setting:**

PDPOP is a co-produced training course aimed at all staff within GP practices to help teams, including administrative, reception, and clinical staff, to feel confident and skilled when interacting with patients who may have personality disorder.

**Method:**

The New World Kirkpatrick Model was used to evaluate the training, through questionnaires pre- training, post-training, at follow-up, and semi-structured interviews.

**Results:**

Evaluation found that practice teams were highly satisfied with PDPOP training and found it relevant, engaging, and useful. Results demonstrated that staff felt more confident and skilled when interacting with patients who may have personality disorder through use of the training’s core concepts. Increased confidence in managing distress, crisis, and participants’ own emotions was associated with reduced impact on staff at interview. Practices also demonstrated action taken post-training to reduce dependency on primary care services.

**Conclusion:**

By including lived-experience trainers and introducing core concepts, PDPOP has demonstrated a sustained positive impact on primary care teams. Further expansion of this type of training may help to increase the confidence of healthcare staff in delivering care to patients with personality disorder and similar complex emotional needs.

## How this fits in

Primary care staff can feel overwhelmed when working with patients with personality disorder. A whole-team training programme using clinical and lived-experience trainers shows a sustained positive transformation in skills and confidence working with this group.

## Introduction

Personality disorder is common and patients with personality disorder often present frequently or in crisis to primary care.^1^ Despite evidence that the provision of specialised services for those with personality disorder is growing, there remains inconsistency and variability in the quality and availability of these services.^2,3^ Additionally, individuals who fall under the category of having personality disorder have a significantly lower life expectancy than the average population,^4^ higher rates of comorbidity,^5^ poorer quality of life,^6^ and up to 20 times greater risk of suicide.^7^

GPs and primary care services have been identified as playing a crucial role in the care of people with personality disorder, often as the first point of healthcare contact and the route through which to be referred on to specialised services.^7^ Recent literature has demonstrated that experiences of individuals living with a personality disorder, whether diagnosed by a clinician or not, are varied.^8^ Those with a personality disorder frequently face stigma and experience difficulties when accessing health care.^8,9^

Correspondingly, primary care staff often describe patients with personality disorder as being ‘difficult’ or ‘complicated’, leaving staff feeling demoralised, incompetent, hurt, or angry.^10^ The need for system-wide training and support has been highlighted to improve staff skill and care delivery.^11^ In response, the Personality Disorder Positive Outcomes Programme (PDPOP) has been developed to meet this need.

### Overview of the Personality Disorder Positive Outcomes Programme

PDPOP is a training course aimed at staff working within GP practices. The main objective of PDPOP training is to help all members of GP teams, including administrative, reception, and clinical staff to feel confident and skilled when interacting with patients who may have personality disorder. Training is co-produced and facilitated by clinical trainers. The trainers are individuals from a mixture of clinical backgrounds, including GPs and therapists and lived-experience trainers; individuals with lived experience of personality disorder. All lived-experience trainers have completed treatment in a specialist treatment service and received training that enables them to reflect on their past experiences in PDPOP sessions without becoming activated in the present. Please see the Supplementary Figures S1 and S2 for further details about the recruitment and involvement of lived-experience trainers.

The training programme consists of whole-team training for all practice staff of either 4 hours or 2.5 hours. Follow-on support is provided for a lead GP in each practice consisting of a debrief and action planning session with a GP clinical trainer, a follow-on training module, and a residential symposium (1 evening and 1 full day), which includes facilitated skills workshops and simulated learning. Please see the Supplementary Figures S3, S4 and S5 for further information about the components of the training and residential symposium.

**Figure 1.**
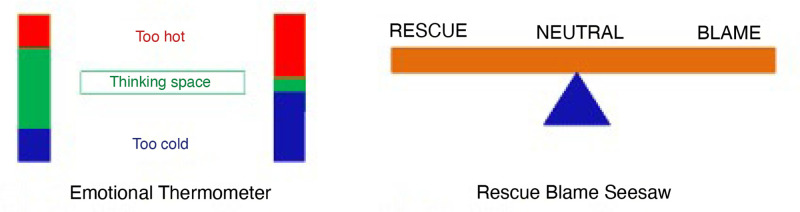
Emotional Thermometer (originally developed within Oxford Health NHS Foundation Trust’s Complex Needs Service based on concepts from mentalisation-based therapy^17^ and Rescue Blame Seesaw (based on^18,19^) © Rob Schafer and Fiona Blyth 2019 (https://www.pdpop.uk/skills)

Whole-team training is made up of a mix of small group and whole-group discussions, presentations, videos, and action planning. Throughout the training PDPOP introduces core concepts to help give staff a shared language for talking about common issues. These core concepts include unmet need, triggers, the Emotional Thermometer, and Rescue Blame Seesaw (Figure 1).

### Method

Health Innovation Oxford and Thames Valley was commissioned to conduct an independent evaluation of the PDPOP training. The New World Kirkpatrick Model^12^ was used as a framework for the evaluation. This report focuses on levels 3 and 4 of the model.

Level 3 of the evaluation sought to determine the behavioural changes that resulted from PDPOP training through identification of critical behaviours and required drivers. Critical behaviours are specific actions which, if performed consistently in practice, will have the biggest impact on results after training. Required drivers are described as the processes and systems that reinforce and encourage critical behaviours.^12^

The intended purpose of level 4 evaluation is to measure one singular outcome that pertains to the purpose of the organisation undertaking training. However, relating a single training course to a high-level organisational outcome can be problematic, and so results may be measured through leading indicators. Leading indicators are defined as the observations in practice that suggest critical behaviours are on track to have a positive impact.^12^

The following leading indicators were defined by the evaluation team and training leads through the intended results and outcomes of the training before training took place:

- staff to feel confident and skilled when interacting with patients who may have personality disorder;
- reduced impact on staff; and
- recognition and reduction of dependency for patients on primary care services and staff.

The evaluation consisted of questionnaires administered pre-training, post-training, and at follow-up (6–8 weeks post-training) and semi-structured interviews of practice staff at follow-up, and GP leads at 4–6 months post-training.

The questionnaires contained a unique identifier field that enabled matched responses over time and contained repeated measure 5-point Likert items, (strongly disagree to strongly agree), and free-text questions. Analysis of Likert responses was conducted using SPSS (version 29.0), analysis of free-text responses and interviews were conducted using NVivo (version 14) and thematic analysis.^13^

*T*-test analysis was undertaken to determine whether there were significant differences between the pre-training and post-training Likert item responses of all participants against those of follow-up participants. A significant difference (*P* = 0.02) was found for one item: ‘I have days where I feel rubbish because of difficult interactions with patients’. For this item, follow-up participants had higher agreement responses pre-training on average than all participants.

There was no significant difference (*P*>0.05) between all participant and follow-up participant responses for all other items. Therefore, it can be assumed that follow-up responses are representative of all training participants for these items.

All interviews were recorded and informed consent obtained before interview. Once transcripts of interviews were verified as accurate, recordings were deleted, and transcripts anonymised through allocation of a participant number.

Likert item responses include only matched responses for comparisons of pre- and post-training and of pre-training and follow-up. Free-text question responses from all participants (not just matched responses) for both the post-training and follow-up questionnaires have been included alongside interview responses.

Semi-structured interviews were also conducted by the evaluation team with lived-experience trainers to gain further insight into the experience of delivering the training. These interviews were not recorded, but detailed contemporaneous notes were taken and then transcribed. A summary of the themes of these interviews was sent to all lived-experience trainers who participated for sense checking. The themes from these interviews can be found in the Supplementary Boxes S1, S2, and S3.

### Recruitment and participation

Training was delivered to 10 GP practice teams across Southeast England. Practices were recruited to training via a self-sign-up process, following advertisement through promotional flyers distributed to GP training hubs, GP training scheme programme directors, and assistant directors. The training was delivered free of charge to the practices; however, participation in the evaluation programme was an expectation of practices stated at the recruitment stage.

## Results

All 10 practices completed the initial training, five practices received the 4-hour training and five received the 2.5-hour training. One practice withdrew from follow-up activities, including follow-up evaluation. The remaining nine practices all took part in follow-up activities and follow-up evaluation.

A total of 386 staff were trained across the 10 practices. Training group size for practices varied from 89 participants to 12 participants. The most represented job roles of those who attended training were administration (23%) and GP (21%) followed by receptionists (16%) and nurses (13%). Representation of job roles across the 10 practices was similar for GPs, receptionists, nurses, administration, and management staff, with less representation across practices for other roles (Table 1). Responses to follow-up were proportional to training attendance with regard to job role. The largest participant job roles at follow-up were GP (*n* = 18), administration (*n* = 17), and receptionist (*n* = 17).

**Table 1.** Participant job roles and distribution across practices

Job role	Number of participants (%)	Number of practices attended from
Administration	87 (23)	9
GP	80 (21)	10
Receptionist	61 (16)	10
Nurse	50 (13)	10
Management	27 (7)	9
Healthcare assistant	23 (6)	7
Pharmacist	19 (5)	7
Social prescriber	4%	6
Mental health practitioner	3%	5
Paramedic	2%	5
Other (physician associate, phlebotomist, student paramedic, medical student, student GP)	1%	4

All 10 practices completed pre- and post-training questionnaires resulting in 331 matched pre- and post-training questionnaires (86% matched response rate).

Of the nine practices who continued with follow-up activities, eight practices completed follow-up questionnaires resulting in 70 matched pre-training and follow-up questionnaires (22% matched response rate).

Nine practice staff agreed to interviews from four practices. The staff interviewed came from a variety of roles, including GPs, mental health practitioners, administrators, and management roles. Seven of the nine GP leads participated in interviews.

There were no significant differences found in the evaluation of the training between participants who received the 4-hour training and participants who received the 2.5-hour training.

A significant difference (P = 0.02) was found for one item: ‘I have days where I feel rubbish because of difficult interactions with patients’. For this item, follow-up participants had higher agreement responses pre-training on average than all participants. There was no significant difference (P>0.05) between all participant and follow-up participant responses for all other items. Therefore, it can be assumed that follow-up responses are representative of all training participants for these items.

### Reaction and learning (levels 1 and 2)

Participant responses to the five items relating to relevance and satisfaction in the post-training questionnaire demonstrates that the training was highly relevant and that participants were highly satisfied with the training (Table 2).

**Table 2.** Participant relevance and satisfaction responses

	Participants who agree or strongly agree, %
I enjoyed the training	98
The training was useful	97
There was enough time for discussion	93
The training was pitched at the right level	95
I would recommend this training to another GP practice	97

Overwhelmingly, the participants valued engagement with the lived-experience trainers, with 225 participants referencing this as the biggest impact from the training. The opportunity for engagement and discussion with the lived-experience trainers and time to ask questions was identified as impactful by participants:

*‘The two* [lived-experience trainers] *who kindly allowed us to ask questions, I felt I would never have been able to ask normally, and I surprised myself of how anxious I was delving into their personal life and I thank them for the courage and help they have both given us today.’* (Manager)

Participant responses to the items relating to knowledge show a significant increase post-training and retained knowledge at follow-up, Table 3.

**Table 3.** Participant knowledge responses

	Participants who agree or strongly agree, %		
	Pre-training	Post-training	Follow-up
I have a **good understanding** of what **personality disorder is**	44	95	94
I can **recognise** when a patient **might have a personality disorder**	46	90	94
I have a good understanding of the challenges other practice staff face when dealing with patients with personality disorder	49	95	96

Participants showed changes in attitude towards patients, particularly those who may present in a confrontational or aggressive manner. Participants also reported that the training had provided them with a better understanding of why patients with personality disorder may behave in the ways that they do and acknowledging and trying to understand triggers:

*‘Understanding there are multiple factors why patients behave in certain ways and try to help and support where we can*.’ (Nurse)

Participants also highlighted the impact of the training through the whole-practice approach. They commented on being able to relate to other staff experience as well as hearing the experience of those in different roles and being more aware of the challenges others experience:

*‘Hearing thoughts of my colleagues and their experiences. Working together to come up with strategies to better support each other and patients.’* (GP)

Several participants highlighted the impact of the training as a whole team as being able to work better together through having a shared language:

*‘It has had a very positive effect on us. It has made everyone more empathetic and understanding. It has also been very helpful in giving us the vocabulary to discuss and manage patients with personality disorder and similar complex emotional issues.’* (Manager)

### Behaviour and results (levels 3 and 4)

#### Critical behaviours and required drivers

Findings are taken from the follow-up questionnaire responses and interviews with practice staff and GP leads. The main themes in participant responses that evidenced critical behaviours were self-reflection, application of the concepts taught in day-to-day work, and changes in thinking and approach to patients:

*‘I have used some of the tools with patients, especially the emotional thermometer. I have also been able to better recognise when a patient is in the "red zone" and adjust my consulting accordingly.’* (GP)

*‘Taking the time to listen to a patient’s concerns regarding a health complaint and offering suggestions of ways to help themselves, without "rescuing" completely.’* (Nurse)

*‘Becoming aware of care with follow-up, of promoting agency, of encouraging patient to problem solve.’* (GP)

*‘I have learned to treat each interaction with a patient as a new event and not let previous interactions colour my opinions/expectations of patients.’* (Manager)

Required drivers were evaluated as the changes made by participants as a team that act to reinforce and encourage the training. The main themes in participant responses that evidenced required drivers were meeting as a team, revisiting training in conversation, reminding each other, and challenging old behaviours:

*‘I have heard team members discussing complex patients much more often and considering new ways to approach situations rather than slipping into expressing frustration/anger.’* (Unknown role)

*‘We have the certificate displayed in reception and a patient commented on how good it was to see we are a friendly PD-friendly practice.’* (Receptionist)

*‘I have a better understanding of personality disorders and how it can affect the patient. As a team we try to remind less tolerant members of staff of the training.’ (*Admin)

### Leading indicators

#### Staff to feel confident and skilled when interacting with patients who may have personality disorder

The evaluation found that participants felt more confident and skilled when interacting with patients who may have personality disorder than before the training. Participant responses to Likert items around confidence demonstrated that the training increased their confidence in dealing with patients presenting in crisis post-training and that this confidence was retained at follow-up (Table 4).

**Table 4.** Participant confidence responses

	Participants who agree or strongly agree, %		
	Pre-training	Post-training	Follow-up
I would **feel confident** dealing with a patient **presenting in crisis** with expressions of desperation, self-harm, or thoughts of self-harm	33	78	73
I can manage my own emotional reactions to patients who present in an emotional crisis	66	^a^	87
My team responds effectively to patients who present in an emotional crisis	62	^a^	91

^a^Not asked post-training

The items regarding managing participants’ own emotions and how their team responds to patents in crisis were repeated only at follow-up. Although participants rated these items positively pre-training, responses at follow-up show an increased confidence for both these items.

Increased confidence was reported across job roles by both those working clinically and non-clinically:

*‘I think I could deal with someone with a personality disorder more confidently. After listening to* [the lived-experience trainers’] *point of view, seeing two sides has help me understand how they are feeling.’* (Admin)

*‘More confidence facing difficult conversations with patients and knowing that it can help them.’* (Nurse)

#### Managing emotional reactions using the concepts of the Emotional Thermometer and Rescue Blame Seesaw

Many participants described how feeling more confident to manage crisis and distress also meant being able to better manage their own emotions:

*‘I might even glance at my little pictures of a seesaw and the thermometer and think okay. Right now they’re in the red and there’s no point me jumping in as well. It’s just giving a framework, I think that when you feel yourself, when it’s going down a very emotional sort of route and it’s being compassionate but at the same time let’s try and steer it back to do some therapeutic work.*’ (GP lead)

*‘The emotional thermometer, which is really helpful for communicating as I say, not just patients with personality disorders but for all patients and also communicating amongst the team a little bit.’* (GP lead*)*

*‘Emotional seesaw has been very useful, the image has stuck in my mind ever since the training. We as a team also refer to it quite a lot and discuss how to stay neutral.’* (Manager)

*‘Recognition that with one patient I have been flipping between blame and rescue and I should try to be somewhere in the middle.’* (GP)

*‘I don’t get pulled into the rescuer role, because although I am a natural rescuer…. I now have a better understanding that often rescuing is not really helpful, so making sure you’re pushing back a little bit and it’s almost as though the course has given you permission to push back and say, "I’m not gonna take that one from you”.’* (GP lead)

#### Reduced impact on staff

Participants’ responses to increased confidence and feeling they can better manage their own emotions were linked to the leading indicator of reduced impact on staff, such as feeling less like they were doing things wrong or doing a bad job, feeling more able to help patients and better able to leave ‘work things’ at work.

 Follow-up participant agreement to the Likert item ’I have days where I feel rubbish because of difficult interactions with patients’ reduced by 10%. This topic was further explored at interview where participants reflected on how, since the training, their increased understanding of personality disorder had led to confidence in being able to not take things personally and feeling empowered:

*‘And you could think actually that wasn’t about me. It was about maybe something’s going on in their life and I was just the front person that happened to be there at the time.’* (Manager)

*‘This training was wonderful and very thought-provoking, in a positive way. I feel I have a lot of empathy; however, this training made me realise that coming into contact with a person that displayed aggressive, repetitive, or needy traits are to be listened to more in-depth. And could be suffering from personality disorder and asking for help in the only way they know how.’* (Admin)

*‘Significant increase in understandings of these patients/less stress from myself when situations can make me feel powerless, I remind myself that staying the neutral container is what is going to help my patients the most in the long run.’* (GP)

#### Recognition and reduction of dependency for patients on primary care services and staff

Agreement responses to the Likert item ’I have patients who I think have become dependent on me in a way that is not helpful for them’ were similar throughout the evaluation (pre-training 21%, post-training 27%, follow-up 23%). However, acknowledgement of dependency and changes to reduce this was a strong theme at follow-up. Participants spoke about the challenges related to providing care that has continuity and builds a relationship, while ensuring that patients do not become dependent on one individual, as well as having increased recognition of when a practice might be facilitating dependency by scheduling repeated reviews with no clear agenda:

*‘I think it’s recognising. “Hang on. Why are we why you coming to see me again?” You know it’s recognising that. It’s funny, cause you kind of know it, but you don’t. Before I kind of knew it, but I didn’t. I didn’t sort of recognise. Recognise it in terms of unmet need, perhaps.’* (GP lead)

*‘And so we’ve said to GPs as a whole that that’s not how we should be doing it and we shouldn’t be just a rolling programme of reviews. Unless there is a very clear indication to do so. And I think that part of having that conversation together as a group, which we did as a sort of a GP meeting, we then talked about that again. I think getting it out on the table for us all to discuss around those reviews and why we find it difficult sometimes.’* (GP lead)

##### Improved understanding of the limitations of medication

Many GP participants spoke about a change in the way they viewed medication for patients with personality disorder, describing how they had learnt the value of a prescription for patients and that this may represent ‘*care*’, acknowledgement, and validation for someone. Highlighting that if medication is not the answer, this needs to be replaced with another option, and a clear and open conversation about the limited value — and the potential harms — of prescription medication. Examples were given of more open conversations with patients about medication and feeling more confident when addressing this issue:

*‘At the start they were wanting more medication, even though they are on quite a bit already. And I managed to turn that around and sort of say, well, and we’ve came up with a deprescribing plan for them. So that was quite good this week.’* (GP lead)

##### Change in consultation style

A change in the style of consultations was a theme across GP responses. This often centred around taking a more structured approach, focusing on one issue and not trying to ‘*fix*’ all problems, even those that may not be health related. Also, not trying to ‘*cure*’ patients and finding a productive way to say no to requests that aren’t appropriate in a non-inflammatory way:

*‘One of my patients with strong suspicion of PD is very demanding and calls several times a week, writes letters, offers flowers when* [they] *want something out of us I have responded less immediately to* [their] *queries and offered* [them] *some time to talk about* [their] *past issues which has shared another light of our doctor–patient relationship — it has created a safe space with less stress for both of us and emotions can now be expressed and contained.’* (GP)

*‘Been clearer with one of my patients about the length of time we have together in appointments. Helping* [them] *to remember this in consultations. Initially* [they] *were really upset by this but have responded more and more positively. I’ve been quite surprised how easily* [they] *have allowed me to park topics and direct* [them] *back to our set aim.’* (GP)

*‘Confidence. Really. With dealing with complex consultations with patients with personality disorder, obviously, but also I’d say any sort of more challenging, consultations, where perhaps needs to be thinking more about boundaries and thinking about patients, unmet needs and things. So yeah I think it’s just given me much more confidence actually with those sort of difficult consultations.’* (GP lead)

*‘And then having the training has cemented how you know … Being clear, being firm is the right thing to do. And it’s not that we’re being bad doctors. And then … because we see the positive effect of it, that enables us to feel better about doing that and continuing to do that to set those boundaries. And you feel more positive because it’s the right thing to do and it’s having less emotional effect on you as well.’* (GP lead)

*‘Not coming away feeling that you’ve achieved nothing, you haven’t helped them and the person’s probably worse off than where you were before, but you can just pick one route to go down and say okay what you mentioned that let’s focus on that.’* (GP lead)

Some GP leads reported that more focused consultations have resulted in shorter and less frequent consultation times:

*‘And it’s already making a difference as all of my patients just the change in tack rather than the half an hour appointments I got off the phone in 10 minutes the other day, which is unheard of.’* (GP lead)

*‘Yeah, and less frequent … And just saying, right, we’re gonna focus on point A. But we’ll do point B next time and next time is going to be then. And I’ve only got 10 minutes, so we’re gonna stick with 10 minutes. And being very clear from the beginning. Lessens the need for them to phone up daily, saying I’m still in a panic, so it’s definitely lessened.’* (GP lead)

Despite overwhelmingly positive feedback about the training, in interviews some participants highlighted that there were challenges to putting the training into practice. They frequently expressed that these were general challenges faced in primary care and not specific to the PDPOP training. Challenges included high staff turnover, pressures and competing demands, potential scepticism of staff, and a lack of services to refer on to for further support.

## Discussion

### Summary

The evaluation of the PDPOP training has found that participants were highly satisfied with the training and found it to be relevant, engaging, and useful. Results demonstrated that participants had greater knowledge about personality disorder and demonstrated shifts in attitude towards including, acknowledging, and trying to understand patients, with greater empathy and compassion.

Success of the training was demonstrated through critical behaviours and required drivers in practice being reported by participant responses at follow-up, resulting in leading indicators being present. Evaluation results show that PDPOP training increases staff feelings of confidence and skill working with patients who may have personality disorder, through use of the training’s core concepts and increased confidence in managing distress, crisis, and participants’ own emotions.

Increased confidence was associated with reduced impact on staff thematically at interview where participants spoke about not taking things personally and feeling empowered. Specifically for GPs, learning around medication and structured consultation styles led to increased confidence, feeling less overwhelmed, and better time management. GPs felt more positive after consultations that they were doing a better job and were less emotionally affected.

The training did not quantitatively show an increase in participants’ recognition of patients who may have developed unhelpful doctor dependency. However, follow-up questionnaires and interviews revealed evidence of action taken post-training to reduce dependency as a result of the training.

### Strengths and limitations

The evaluation was conducted by an independent team to the training team and captured a cross-disciplinary range of training participant views. The use of matched response questionnaires at three time points alongside interviews at two further time points enabled in depth exploration of the effect of the training and how this was sustained within GP practices for a range a staff. This was supported through the use of the New World Kirkpatrick Model as a validated framework for training evaluation, which facilitated evaluation beyond immediate satisfaction to effects and changes made in practice.

Potential limitations of the evaluation are the lower response rate to the follow-up questionnaire and that practice staff (non-GP leads) interviews were across four out of nine of the GP practices. Another limitation may be that responses from GP leads, who individually had further follow-on support, have been reported alongside other practice staff. Additionally, the perspective of patients from the trained GP practices was out of scope of the present evaluation; however, further longitudinal evaluations of the PDPOP training should consider how to evaluate the effect of the training for those accessing GP services.

### Comparison with existing literature

PDPOP embodies the three key aspects identified within the literature of effective training for health professionals of co-production, communicating a psychological model (four key concepts), and teaching participants skills they can use within their work and interactions.^14^ Evaluation of PDPOP has shown that training around personality disorder has a positive effect in primary care staff, both in clinical and non-clinical roles, which is novel when compared with previous recent literature evaluating comparable training such as the Knowledge and Understanding Framework (KUF), which has focused on delivery to mental health professionals.^15,16^

### Implications for practice

In conclusion, PDPOP is a training programme that trains whole-practice teams together and is co-produced and delivered by lived-experience trainers and clinical trainers. By including lived-experience trainers and introducing core concepts, PDPOP has demonstrated a sustained positive impact on primary care teams to support them to meet the health needs of patients with personality disorder and similar complex emotional needs. Further expansion of this type of training may help to increase the confidence of healthcare staff in delivering care to this patient group.

## Data

The dataset relied on in this article is available from the corresponding author on reasonable request.
